# Treatment of dermal ulcer with autologous fibrin glue: Two case reports of an exploratory prospective pilot study

**DOI:** 10.1097/MD.0000000000036134

**Published:** 2023-11-17

**Authors:** Shinichiro Kawamoto, Eriko Shinkawa, Susumu Fujiwara, Yoshiko Oda, Haruki Jimbo, Eiji Nakano, Takeshi Fukumoto, Ryusuke Ono, Takahiro Yasuda, Hironobu Minami

**Affiliations:** a Department of Transfusion Medicine and Cell Therapy, Kobe University Hospital, Kobe, Hyogo, Japan; b Division of Medical Oncology/Hematology, Department of Medicine, Kobe University Hospital Graduate School of Medicine, Kobe, Hyogo, Japan; c Department of Dermatology, Kobe University Hospital, Kobe, Hyogo, Japan; d Department of Dermatology, Hyogo Cancer Center, Kobe, Hyogo, Japan; e Division of Medical Devices and Systems, Department of Medical Devices, Kobe University Graduate School of Medicine, Kobe, Hyogo, Japan; f Device Development Promotion Division, Advanced Medical-Engineering Development Center, Kobe University, Kobe, Hyogo, Japan.

**Keywords:** autologous blood donation, autologous fibrin glue, bFGF, refractory skin ulcer, scleroderma, skin transplantation, trauma

## Abstract

**Introduction:**

The healing of recurrent and refractory skin ulcers requires a long time, during which there is risk of infection, and hospital admission is occasionally required for surgical or daily conservative treatment. Therefore, the development of promising treatments that promote faster, uneventful healing is a must. Composed of cryoprecipitate and thrombin, fibrin glue has a history of surgical use for preventing bleeding and spinal fluid leakage. Moreover, in-house cryoprecipitates contain higher concentrations of coagulation factors and cytokines that may enhance wound healing than commercially available products. However, the efficacy of completely autologous fibrin glue (AFG) in tissue repair has not yet been fully demonstrated.

**Patient concerns:**

This study aimed to evaluate the efficacy of AFG in the treatment of refractory skin ulcers in comparison with the conventional treatment.

**Diagnosis:**

Two patients with skin ulcer on their lower extremities due to trauma or scleroderma who showed resistance to conventional treatment were included in the study. Both study participants were diagnosed with refractory skin ulcer and were ineligible for autologous skin transplantation.

**Interventions:**

AFG was prepared following autologous blood donation using a Cryoseal^®^ system. Subsequently, AFG was administered to 50% of the area of each ulcer and observed for 4 weeks in comparison with recombinant basic fibroblast growth factor with bucladesine sodium treatment that was administered to the rest of the ulcer.

**Outcomes:**

The skin ulcer after trauma in participant 1 showed better improvement in the AFG-treated area. Although AFG did not show superiority regarding the ulcer area of a patient with scleroderma, it guarded the continuous exudation from the edge of the swollen skin surrounding the ulcer.

**Conclusion:**

AFG showed effective and beneficial results for wound healing of refractory skin ulcer and prevented exudation without any severe adverse events.

## 1. Introduction

Ulcers in the lower extremities occur secondary to multiple diseases and conditions such as venous congestion, injury, infection, and diabetes mellitus, and are often refractory to treatment. The treatment strategies for cutaneous ulcers vary depending on stage;^[1]^ a severe case requires surgical treatment or daily time-consuming management at minimum. Moist conditions are preferable for healing and pain control.^[2]^ However, excessive effusion causes bacterial infections, and recurrent cases can lead to the emergence of antibiotic-resistant bacterial clones, thereby increasing medical expenses and extending the time required to heal.^[3]^ Additionally, the continuous flow of exudate from ulcers impairs patient quality of life as it leads to hypoalbuminemia and edema in the legs and ultimately gait disturbances, especially in elderly patients.

Several cytokines and cell types play a critical role in wound healing and are being targeted by ulcer treatments. Furthermore, the efficacy of heparinized blood containing cytokines for the treatment of refractory skin ulcers has been previously reported.^[4]^ Among these cytokines, basic fibroblast growth factor (bFGF) stimulates the growth of fibroblast cells, and its recombinant formulation is a key drug for conservative treatment in combination with other topical applications.^[5]^ However, the response depends on the patient’s condition; therefore, additional treatment strategies such as skin substitutes and regenerative medicine are being explored.^[6,7]^

Commercially available allogeneic fibrin glue, also known as fibrin sealant, is widely used after surgery to reinforce sutures and topically inhibit blood and spinal fluid leakage.^[8]^ Cryoprecipitate is concentrated from pooled plasma through freezing and moderate thawing at 4 °C, and the frozen stock is used intravenously after thawing for the management of acquired coagulopathy caused by massive bleeding based on enriched fibrinogen and anticoagulant factors.^[9]^ Commercial or in-house prepared cryoprecipitate requires allogeneic or xenogeneic thrombin to form fibrin glue. The CryoSeal^®^ system (Asahi Kasei Medical, Co., Ltd, Tokyo, Japan) is composed of a CS-1 instrument that undergoes programmed freezing and thawing and a CP-3 disposable kit that semiautomatically produces autologous cryoprecipitate and thrombin, also known as autologous fibrin glue (AFG), under sterile conditions.^[10]^ It has been used postsurgically depending on its physical function.^[11,12]^ Although the application of fibrin glues of autologous cryoprecipitate with xenogeneic or allogeneic thrombin for the fixation of skin grafts or as a scaffold for keratinocytes on chronic skin ulcer have been confirmed, the sole effect of the fibrin glue on skin ulcers remains controversial.^[13,14]^ Because AFG is prepared more mildly than commercial products that use ethanol for fractionation, its biological components remain unaltered.^[15]^ Therefore, AFG may have better efficacy in wound healing through its chemical function.

Based on this background, we conducted an exploratory prospective pilot study comparing the treatment of the cases with single refractory cutaneous ulcer using AFG (dermal ulcer treatment with autologous fibrin glue) with the conventional bFGF treatment.

## 2. Methods

### 2.1. Ethics

This study was an exploratory prospective pilot study conducted in Kobe University Hospital in accordance with the Declaration of Helsinki, from September 2019 to August 2022. The protocol was approved by the ethics committee of Kobe University (approved No. C190016) and registered with the Japan Registry of Clinical Trials (No. jRCTs052190052).

### 2.2. Participants

Refractory skin ulcer was defined as lesion that did not respond to or recur after previous standard treatments such like skin graft and bFGF/bucladesine sodium (BS) treatment. The inclusion criteria were as follows: patients aged ≥ 20 years; skin ulcer between 2 cm to 20 cm in diameter due to diseases such as, venous congestion, diabetes mellitus and vasculitis, unaltered by load or other local factors; patient who requires treatment by bFGF and BS. The exclusion criteria were as follows: patients who do not meet the criteria as per guidelines for autologous blood transfusion donation by the Japanese society of autologous blood transfusion; patients using anticoagulants; patients deficient in essential factors of the coagulation system; patients with past history of malignant disease at the region of the ulcer; patients allergic to recombinant bFGF, BS, and ethanol; patients with an irregular shaped ulcer or an ulcer that includes difference in depth of more than 5 mm, which are difficult to be divided equally by a straight baseline; patients with a deep or large ulcer that requires surgical treatment; pregnant, possible pregnant or lactating women, participants of other clinical trials, patients judged as inappropriate by physicians.

The characteristics of participants are shown in Table 1. The transplanted autologous skin in participant 1 necrosed and participant 2 refused autologous skin transplantation because it required admission. Hence, they were registered to the study.

**Table 1:** Characteristics of participants with refractory skin ulcer in the dermal ulcer with autologous fibrin glue study.

	Participant 1	Participant 2
Age, y	56	87
Gender	Male	Female
Cause of ulcer	Trauma	Scleroderma
Area of ulcer before the study (cm^2^)	20	19
Previous treatments	Autologous skin transplantation, bFGF/BS	bFGF/BS

### 2.3. Intervention

**Figure 1.**
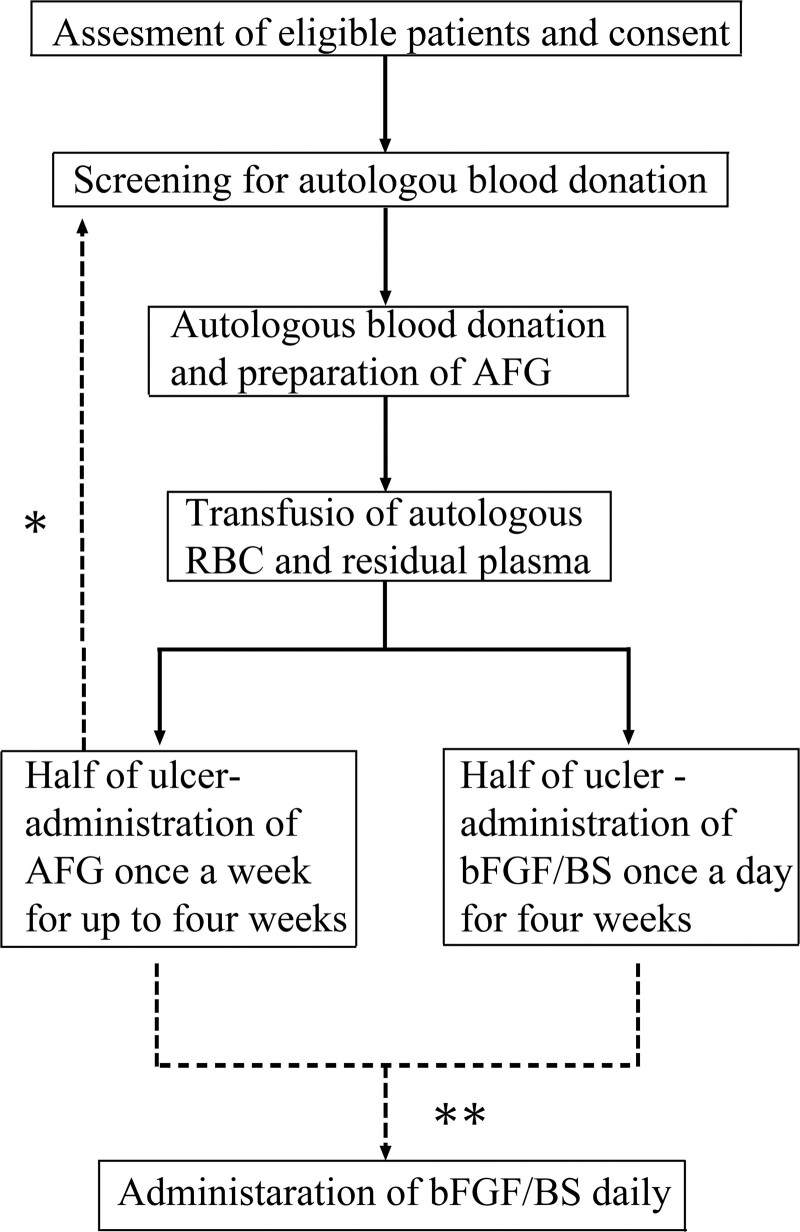
The timeline of the dermal ulcer with autologous fibrin glue study. *Patients with ulcers > 15 cm in diameter and those who require additional treatment, autologous blood donation, and fibrin glue preparation will be treated again. **If the ulcer did not improve completely, the conventional treatment by bFGF will be continued for both areas. AFG = autologous fibrin glue; bFGF = basic fibroblast growth factor; BS = bucladesine sodium; RBC = red blood cell.

After screening for autologous blood donation including laboratory and physiological examination, written informed consent was obtained from participants. The timeline for the study was shown in Figure 1. Donated blood (300–400 mL) was obtained, AFG was prepared immediately from the plasma and stored at −20 °C until the administration. Red blood cells and residual plasma were transfused on the day following autologous blood donation to recover anemia and loss of plasma proteins.

For each participant, the single ulcer was divided into 2 equal areas, treated with either AFG once a week for up to 4 weeks or bFGF with BS daily for 4 weeks.

### 2.4. Measurements

The area of ulcer was calculated using by digital software from the photo image taken from 30 cm distance weekly before and after the treatments. Primary outcome was the improvement in rate of the ulcer surface area healed at 4 weeks after the initial treatment. Statistical analysis was not planned for the results because of its exploratory aim.

## 3. Results

Although the study planned to include 10 patients, only 2 were ultimately registered as the shapes of most ulcers were difficult to divide into 2 equal areas. In the current report, we describe the treatment courses of the 2 registered cases.

### 3.1. Participant 1

**Figure 2.**
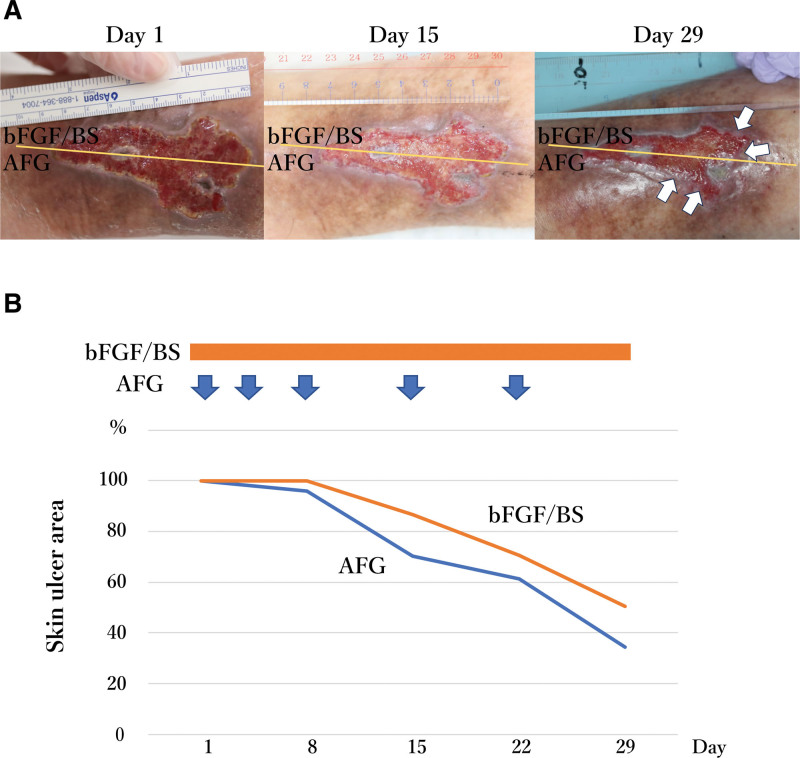
Improvement in AFG- versus bFGF/BS-treated area in participant 1. (A) Morphological changes in the ulcers after treatment. The yellow bar indicates the boundaries of treatments. The white arrows indicate the effectively healed area by each treatment. (B) Improvement in ulcer area. Percentages of unimproved areas are shown with orange or blue line for bFGF/BS and AFG, respectively. The orange bar indicates the daily administered bFGF/BS and the blue arrows indicate the administration of AFG. AFG = autologous fibrin glue; bFGF = basic fibroblast growth factor; BS = bucladesine sodium.

AFG and bFGF/BS were administered to half of the ulcer’s area on day 1. However, the moisture of AFG was immediately absorbed by the gauze, which then dried and detached from the ulcer within 2 days. Therefore, AFG was readministered on day 4 according to the protocol. The BS was spread on a gauze that covered the entire area to avoid moisture absorbance from the AFG, which was administered on Days 8, 15, and 22. The bFGF/BS was administered daily after scrubbing. After 4 weeks, the ulcer treated with AFG showed 66% reduction in area while that of bFGF/BS was 50% reduction (Fig. 2B). Although bFGF/BS treatment was continued for the entire residual ulcer after the study period, complete epithelialization required more than 70 days after the study period.

### 3.2. Participant 2

**Figure 3.**
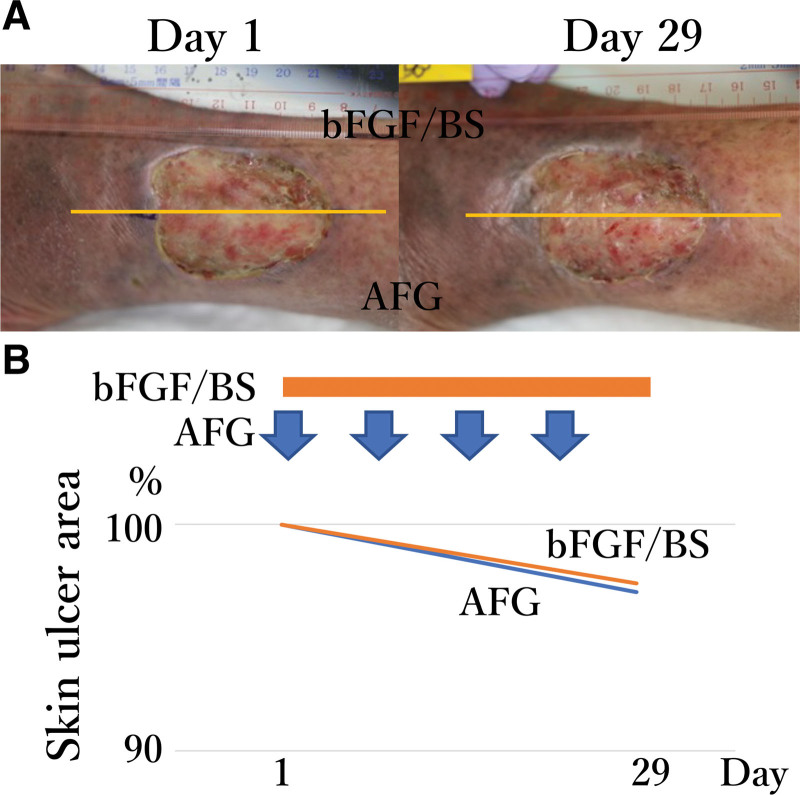
Unchanged ulcer area of participant 2 in both treatment groups. (A) Morphological changes in the ulcer after treatment. The yellow bar indicates the boundaries of treatments. (B) Improvement in the ulcer area. Percentages of unimproved areas are shown with orange or blue line for bFGF/BS and AFG, respectively. The orange bar indicates the daily administered bFGF/BS and the blue arrows indicate the administration of AFG. AFG = autologous fibrin glue; bFGF = basic fibroblast growth factor; BS = bucladesine sodium.

As compared to the initial presentation during enrollment (Fig. 3A), improvements in AFG-and bFGF/BS-treated ulcers area after 4 weeks was 3% and 2.6%, respectively (Fig. 3B). The granulation of the ulcer was observed to be equal, and its depth became shallow. As the skin of the outside ulcer was edematous, continuous outflow of exudate from the edge of the bFGF/BS-treated area was observed after 2 weeks; however, this was not the case for the AFG-treated area.

### 3.3. Adverse events

**Figure 4.**
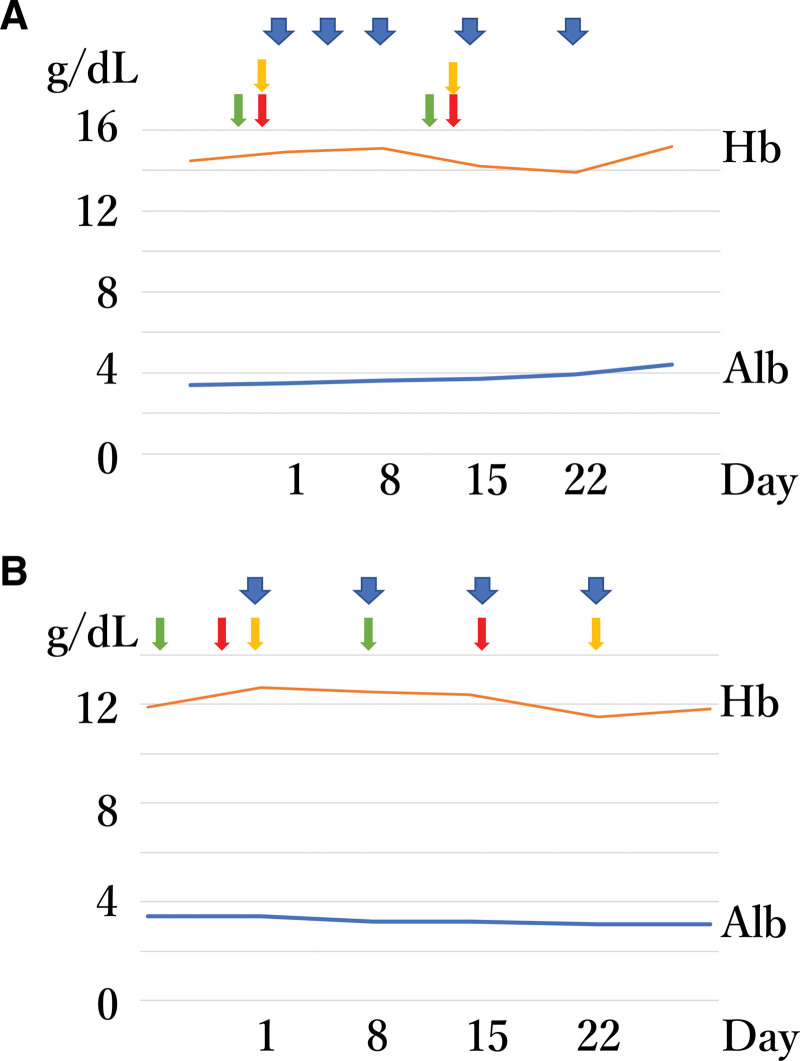
Laboratory examination values before and after the autologous blood donation for AFG preparation and transfusion. (A) Participant 1. (B) Participant 2. Hemoglobin (Hb: orange line) and albumin (Alb: blue line) values were examined weekly. The green arrows indicate autologous blood donation; the red and yellow arrows indicate autologous red blood cell and plasma transfusion that were left-overs of fibrin glue preparation, respectively. The blue arrows indicate AFG administration. AFG = autologous fibrin glue.

There were no symptoms during and after the autologous blood donation in both the participants. In participant 1, anemia was not observed, and hypoalbuminemia at 3.4 g/dL improved during the study (Fig. 4A). However, tingling pain was observed for 1 hour after AFG treatment until the second administration, which was tolerable with the aid of nonsteroidal anti-inflammatory drugs. In participant 2, anemia was not observed after blood donation, and the hypoalbuminemia that had been observed before the study did not worsen (Fig. 4B). Similar to participant 1, tingling pain was observed after AFG treatment for 1 hour until the third administration, which was tolerable with nonsteroidal anti-inflammatory drugs.

## 4. Discussion

The area of ulcer treated with AFG in study participant 1 reduced to 34% within 4 weeks and the exudate in participant 2 was prevented by AFG. Thus, fibrin glue formed by autologous cryoprecipitate and thrombin without autologous skin or keratinocyte grafts, showed good efficacy for wound healing. Autologous RBC and plasma transfusions were considered to prevent anemia and hypoalbuminemia, and the sole adverse event during the treatment was grade 1 pain due to the alcohol contained in the thrombin. The administration of AFG would confer not only direct improvement but also provide safe, supportive effects for refractory skin ulcers patients.

Previous research has explored diverse drugs and materials for tissue regeneration in chronic skin ulcers, reflecting the complexity of the pathogenesis and difficulty of treatment.^[6,7]^ The mechanism of interruption in the blood circulation around the skin ulcer caused by venous congestion, diabetes mellitus and vasculitis are diverse. Additionally, the circumstances of ulcers differ depending on the condition of the patient and the sites of emergence.^[16]^ Therefore, we planned to treat a single ulcer in each patient by dividing them into 2 equal surface areas to make the conditions as similar as possible for the study and control treatments. There is some uncertainty regarding whether the 2 treatments affect each other at the border. However, as the ulcer improved from the peripheral area in participant 1 (Fig. 2), the effect of AFG was likely to be not related to bFGF.

In addition to bFGF, other biological components in blood also play critical roles in wound healing and tissue repair.^[17,18]^ Autologous platelet-rich plasma (PRP) is often used for arthroplasty and has been reported to be effective in the treatment of chronic skin ulcers based on the cytokines released from platelets.^[19,20]^ The epidermal growth factor that is enriched in PRP promotes the growth of cells residing in the legion.^[21]^ As PRP is made from the patient’s blood without chemical procedure, unnecessary immunological response is avoided, and the procedure is safe in terms of pathogenic infection. However, as its viscosity is low, PRP application to an open skin surface requires some skill and sufficient time for immobilization. On the other hand, since AFG coagulates immediately after the administration, almost within a few seconds, it is easier to manipulate compared to PRP with similar safety.

In their study, Takahashi et al confirmed that the concentrations of most coagulation factors in cryoprecipitates prepared by moderate thawing were enriched from the original plasma, and those other than fibrinogen were much higher than that of commercially available products (see Table S1, Supplemental Digital Content, http://links.lww.com/MD/K783, showing the concentrations of biological components in cryoprecipitates).^[22]^ Among these, von Willebrand factor, which binds to platelets and contains platelet microparticles,^[23,24]^ is 10 times more enriched in cryoprecipitates. Since AFG also contains higher concentrations of cytokines than the original plasma, it should have additional biological activities than PRP in wound healing. Although the concentration of chemokines in the AFG has not yet been measured, it is important for the migration of multipotent cells from other parts of the body to damaged lesion.^[17,25]^ Considering the prolonged duration of complete epithelialization after the study period in participant 1, AFG may have affected the entire ulcer area, likely through undetermined biological components such as chemokines. As the cause of scleroderma is fibrotic change induced by fibroblast cells following stimulation by interleukin-6 released from B lymphocytes, the recruitment of mesenchymal stem cells by AFG treatment in combination with the anti-CD20 antibody rituximab may comprise an ideal treatment strategy for study participant 2.^[26]^

The maintenance of aseptic ulcer conditions is critical for resolution. However, this approach requires careful daily treatment and sufficient time. Patients participated in the current study after achieving aseptic ulcer conditions; both AFG and bFGF/BS treatment areas were scrubbed daily before administration to maintain sterile conditions, a procedure that reduces the thickness of the AFG layers. Despite this, weekly administration of AFG showed superior efficacy. As fibronectin, complement, and immunoglobulin are enriched in cryoprecipitate;^[22]^ and the alcohol content in thrombin can suppress the growth of bacteria to maintain aseptic conditions, daily treatment would not be necessary for AFG therapy in the same way after surgery.^[11,12]^ Currently, 3 volumes of AFG of at least 2 mL are prepared from 300 to 400 mL of blood and stored in a freezer for at least 4 weeks after preparation. Therefore, weekly hospital visits are considered sufficient for patients undergoing AFG therapy.

The limitations of current study are the insufficient number of participants included due to the criteria of ulcer shape and the difference in the cause of skin ulcers in the 2 cases that possibly affected the healing response.

A randomized controlled trial with larger number of patients divided into separate treatment groups for each type of refractory skin ulcer, including the complete area of ulcer will help in providing a clear evidence regarding the efficacy of AFG and will further validate the findings of our pilot study.

In conclusion, while autologous blood donation and transfusion must be performed carefully, AFG is expected to offer efficacy not only for treating ulcers and preventing systemic complications but also for improving the quality of life of patients with refractory ulcers.

## Author contributions

**Conceptualization:** Shinichiro Kawamoto.

**Funding acquisition:** Shinichiro Kawamoto.

**Investigation:** Shinichiro Kawamoto, Eriko Shinkawa, Susumu Fujiwara.

**Methodology:** Shinichiro Kawamoto, Eriko Shinkawa, Susumu Fujiwara, Takahiro Yasuda.

**Project administration:** Shinichiro Kawamoto.

**Resources:** Eriko Shinkawa, Susumu Fujiwara, Yoshiko Oda, Haruki Jimbo, Eiji Nakano, Takeshi Fukumoto, Ryusuke Ono.

**Writing—original draft:** Shinichiro Kawamoto.

**Writing – review & editing:** Hironobu Minami.

## Supplementary Material


