# Supplementary material for: Treatment of dermal ulcer with autologous fibrin glue: Two case reports of an exploratory prospective pilot study
Source: Medicine (Baltimore). 2023 Nov 17;102(46):e36134. doi: 10.1097/MD.0000000000036134 (PMC10659605; doi:10.1097/MD.0000000000036134)
Supplement: Supplementary file 1 [file medi-102-e36134-s001.docx]

|  | | Cryoseal | | | product A | product B | |
| --- | --- | --- | --- | --- | --- | --- | --- |
|  |  | Original plasma | | Fibrinogen solution | | | |
| Fibrinogen | (mg/dL) | 263±62 | 1137±265 | | 2260 | | 2455 |
| Antiplasmin | (%) | 105±13 | 101±11 | | 1490 | | 1650 |
| Factor II | (%) | 110.1±11.2 | 204.5±109.3 | | * | | * |
| Factor V | (%) | 105.0±21.4 | 86.2±24.0 | | * | | * |
| Factor VII | (%) | 121.3±33.5 | 160.6±92.9 | | 54.5 | | <5 |
| Factor VIII | (%) | 98.9±67.2 | 574±379 | | <5 | | 160 |
| Factor IX | (%) | 106.0±28.0 | 190.0±176.4 | | <5 | | <5 |
| Factor X | (%) | 110.9±21.1 | 123.3±64.0 | |  | |  |
| Factor XI | (%) | 108.5±36.8 | 145.5±15.9 | | <5.0 | | <5.0 |
| Factor XII | (%) | 119.0±24.5 | 159.1±34.4 | | * | | <5.0 |
| Factor XIII | (%) | 119±39 | 209±60 | | 6500 | | 6450 |
| vWF | (%) | 71±54 | 755±215 | | <200 | | 300 |
| IL-1β | (pg/mL) | <0.2 | <0.6 | | <0.2 | | <0.2 |
| IL-8 | (pg/mL) | <16 | <48 | | <16 | | <16 |
| TGF-β | (pg/mL) | 2124±554 | 2548±517 | | <350 | | <350 |
| G-CSF | (pg/mL) | 28.3±7.1 | ≤58.5 | | ≤19.5 | | ≤19.5 |
| VEGF | (pg/mL) | 23.6±9.7 | 39.6±15.5 | | 12.6 | | 18.5 |
| Fibronectin | (μg/mL) | 209.8±188.8 | 3465.0±1205.8 | | 509.8 | | 6748.5 |
| Vitronectin | (μg/mL) | 72.5±64.8 | 34.3±21.8 | | 1.1 | | 1.3 |
| TNF-α | (pg/mL) | 0.6±0.2 | 1.0±0.4 | | <0.2 | | <0.2 |
| IgG | (mg/dL) | 655±169 | 746±191 | | ≤50 | | ≈50 |
| IgA | (mg/dL) | 157±65 | 173±59 | | ≤10 | | ≤10 |
| IgM | (mg/dL) | 49±35 | 62±39 | | ≤5 | | ≤5 |
| Complement level | (U/mL) | 52±10 | 43±12 | | <5 | | <5 |
| C3 | (mg/dL) | 125±19 | 109±21 | | ≤5 | | ≤5 |
| C4 | (mg/dL) | 28.1±4.9 | 24.1±4.6 | | ≤1.0 | | ≤1.0 |
